# Supplementary material for: Longitudinal Modeling of Age-Dependent Latent Traits with Generalized Additive Latent and Mixed Models
Source: Psychometrika. 2023 Mar 28;88(2):456–86. doi: 10.1007/s11336-023-09910-z (PMC10188428; doi:10.1007/s11336-023-09910-z)
Supplement: Supplementary file 1 — (pdf 462 KB) [file 11336_2023_9910_MOESM1_ESM.pdf]

Online resource 1: Supplementary material to latent response model, Section 5*

Table S1 shows the posterior probability of each possible ordering of the age at maximum across the three cognitive domains. Tables S2 and S3 show Wald type confidence intervals and bootstrap based confidence intervals for all regression coefficients and factor loadings, respectively. Figure S1 shows the empirical distributions of baseline age, measurement intervals, and number of timepoints in the data. In addition to being analyzed in Section 5.1, these data were also used in the simulations in Section 5.2. Figure S2 shows the average estimated trajectories across bootstrap simulations, the true trajectory, as well as all bootstrap estimates. Figure S3 shows a histogram of the sum of effective degrees of freedom of all three trajectories across bootstrap samples.

*This document contains supplementary information to the manuscript "Longitudinal Modeling of Age-Dependent Latent Traits with Generalized Additive Latent and Mixed Models".

Table S1: Probability of each possible ordering of age at maximum for the three cognitive abilities studied in Section 5.1.

Ordering (by increasing age at maximum ability)	Probability
Episodic memory < Executive function < Working memory	1.62 %
Episodic memory < Working memory < Executive function	2.87 %
Executive function < Episodic memory < Working memory	88.4 %
Executive function < Working memory < Episodic memory	6.82 %
Working memory < Episodic memory < Executive function	0.28 %
Working memory < Executive function < Episodic memory	< 0.1 %

Table S2: Regression coefficients in latent response model from Section 5. The second column contains point estimates and asymptotic standard errors and the third column contains bootstrap estimates and standard errors.

Parameter	Estimate (95% CI)	Bootstrap (95% CI)
Episodic memory		
CVLT trial 1 β_{t1}	-0.26 (-0.29, -0.23)	-0.25 (-0.28, -0.22)
CVLT trial 2 β_{t2}	0.74 (0.71, 0.78)	0.77 (0.73, 0.81)
CVLT trial 3 β_{t3}	1.42 (1.37, 1.47)	1.46 (1.40, 1.50)
CVLT trial 4 β_{t4}	1.84 (1.78, 1.89)	1.88 (1.75, 1.88)
CVLT trial 5 β_{t5}	2.17 (2.11, 2.24)	2.22 (2.16, 2.28)
CVLT 5 min delay β_{t6}	1.62 (1.56, 1.67)	1.66 (1.60, 1.72)
CVLT 30 min delay β_{t7}	1.81 (1.75, 1.88)	1.87 (1.80, 1.93)
Working memory		
Digit span backward β_{t8}	-0.39 (-0.41, -0.36)	-0.39 (-0.41, -0.36)
Digit span forward β_{t9}	0.29 (0.26, 0.31)	0.29 (0.26, 0.31)
Executive function (units = seconds)		
Stroop 1 β_{t10}	32.2 (31.8, 32.5)	32.1 (31.7, 32.4)
Stroop 2 β_{t11}	23.3 (22.9, 23.6)	23.2 (22.9, 23.6)
Stroop 3 β_{t12}	58.3 (57.6, 59.0)	58.3 (57.6, 58.9)
Stroop 4 β_{t13}	65.4 (64.7, 66.1)	65.3 (64.7, 66.0)
Retest effects		
CVLT β_{r1}	0.11 (0.08, 0.15)	0.12 (0.08, 0.15)
Digit span β_{r2}	0.08 (0.05, 0.11)	0.08 (0.05, 0.11)
Stroop conditions 1 and 2 β_{r3}	-1.24 (-0.79, -1.69)	-1.23 (-0.83, -1.65)
Stroop conditions 3 and 4 β_{r4}	-2.21 (-1.51, -2.90)	-2.24 (-1.50, -2.87)

Table S3: Factor loadings in latent response model from Section 5. The second column contains point estimates and asymptotic standard errors and the third column contains bootstrap estimates and standard errors. Fixed factor loadings are omitted since their uncertainty by definition is zero.

Parameter	Estimate (95% CI)	Bootstrap (95% CI)
Episodic memory		
CVLT trial 2 λ_{12}	1.79 (1.72, 1.85)	1.77 (1.68, 1.85)
CVLT trial 3 λ_{13}	2.44 (2.35, 2.52)	2.40 (2.28, 2.53)
CVLT trial 4 λ_{14}	2.76 (2.66, 2.87)	2.72 (2.59, 2.86)
CVLT trial 5 λ_{15}	3.02 (2.90, 3.13)	2.97 (2.83, 3.13)
CVLT 5 min delay λ_{16}	3.04 (2.93, 3.15)	2.99 (2.85, 3.13)
CVLT 30 min delay λ_{17}	3.22 (3.10, 3.34)	3.17 (3.02, 3.33)
Working memory		
Digit span forward λ_{22}	0.96 (0.90, 1.02)	0.96 (0.89, 1.03)
Executive function (units = seconds)		
Stroop 2 λ_{32}	-3.96 (3.51, 4.41)	-3.95 (3.50, 4.46)
Stroop 3 λ_{33}	-20.2 (19.3, 21.1)	-21.2 (19.8, 22.7)
Stroop 4 λ_{34}	-21.7 (20.7, 22.7)	-22.8 (21.3, 24.4)

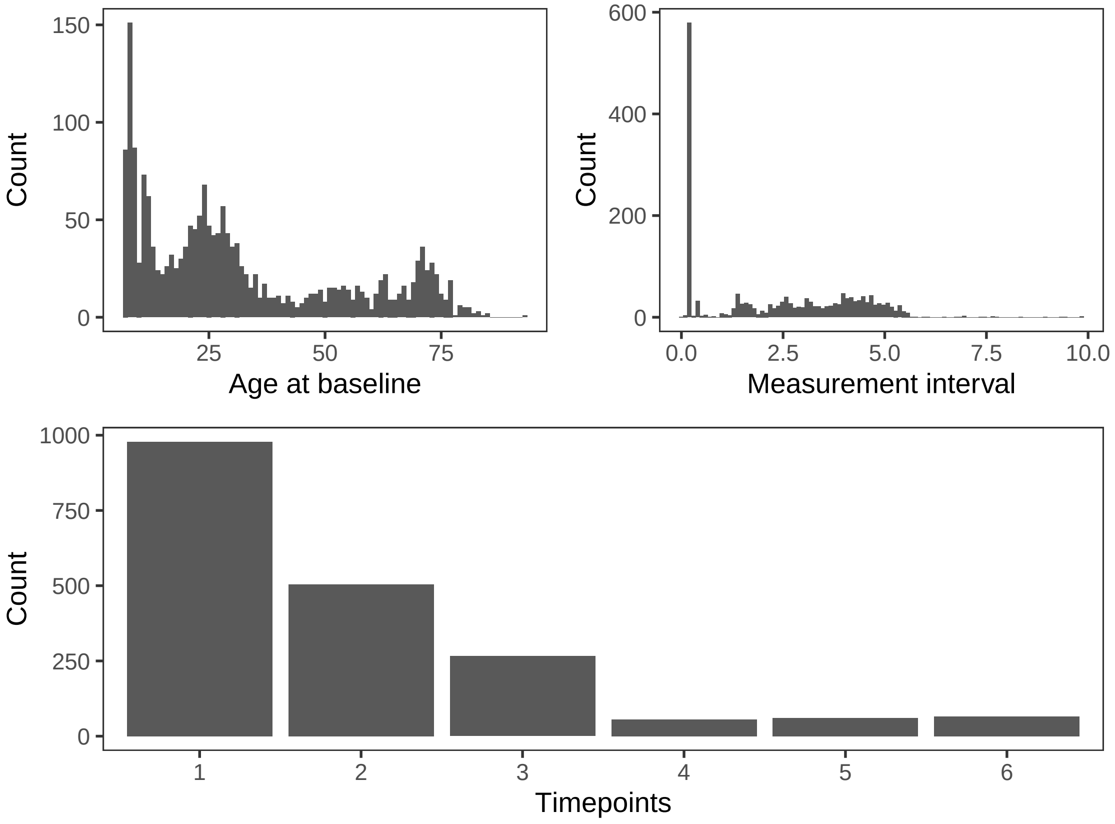

Figure S1: **Age, interval, and timepoint distributions in data.** The top left plot shows a histogram of baseline age and the top right plot a histogram of times between measurements used in the data used in Section 5. The bottom plot shows the numbers of timepoints per participant.

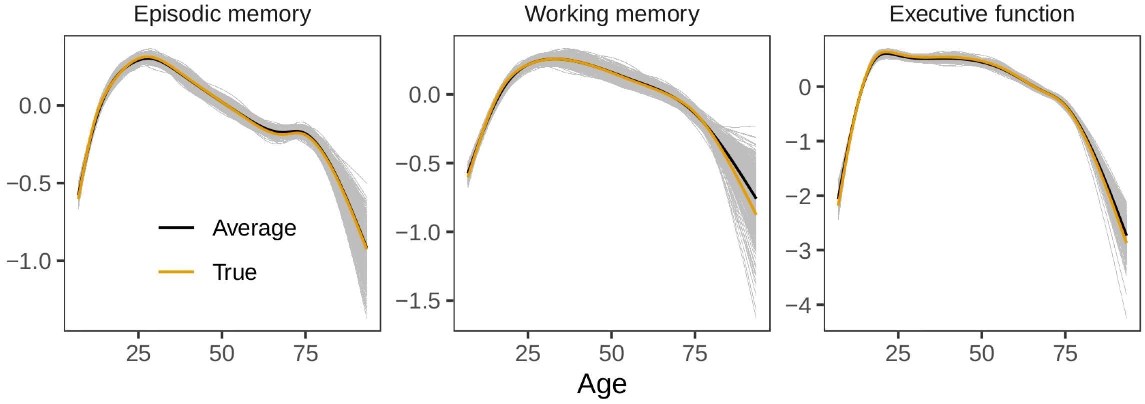

Figure S2: **Results of simulation study.** Average estimates across bootstrap samples plotted together with the true curve for each cognitive domain. Gray curves in the background show all bootstrap estimates.

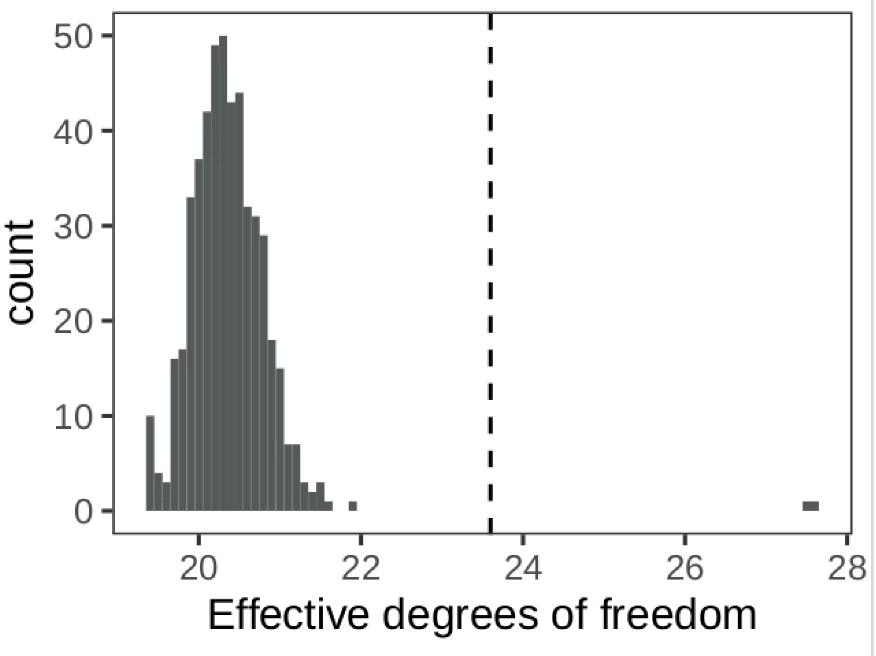

Figure S3: **Effective degrees of freedom of bootstrap estimates.** Histogram of total effective degrees of freedom for the three curves across all 500 bootstrap samples. The dashed line shows the effective degrees of freedom of the model estimates, which was used as a data generating mechanism.
