# Supplementary material for: Longitudinal Modeling of Age-Dependent Latent Traits with Generalized Additive Latent and Mixed Models
Source: Psychometrika. 2023 Mar 28;88(2):456–86. doi: 10.1007/s11336-023-09910-z (PMC10188428; doi:10.1007/s11336-023-09910-z)
Supplement: Supplementary file 2 — (pdf 265 KB) [file 11336_2023_9910_MOESM2_ESM.pdf]

Online resource 2: Supplementary material to latent covariates model, Section 6*

Table S1 shows item intercepts for the latent covariates model presented in Section 6.1. Figure S1 shows the distributions of baseline age, measurement intervals, and number of timepoints per participant, used in the simulations in Section 6.2. Figure S2 visualizes the trajectories under the interaction effects assumed in the simulation studies. Figure S3 shows bias of estimated factor loadings.

*This document contains supplementary information to the manuscript "Longitudinal Modeling of Age-Dependent Latent Traits with Generalized Additive Latent and Mixed Models".

Table S1: Item intercepts in model of hippocampal volume and socioeconomic status.

Parameter	Estimate	SE	Units
Item intercepts			
Father's education, β_{s1}	2.81	0.00684	log(years)
Mother's education, β_{s2}	2.81	0.00671	log(years)
Education, β_{s3}	2.81	0.0053	log(years)
Father's income, β_{s4}	13.2	0.0399	log(NOK)
Mother's income, β_{s5}	13.2	0.0368	log(NOK)
Income, β_{s6}	13.1	0.0367	log(NOK)

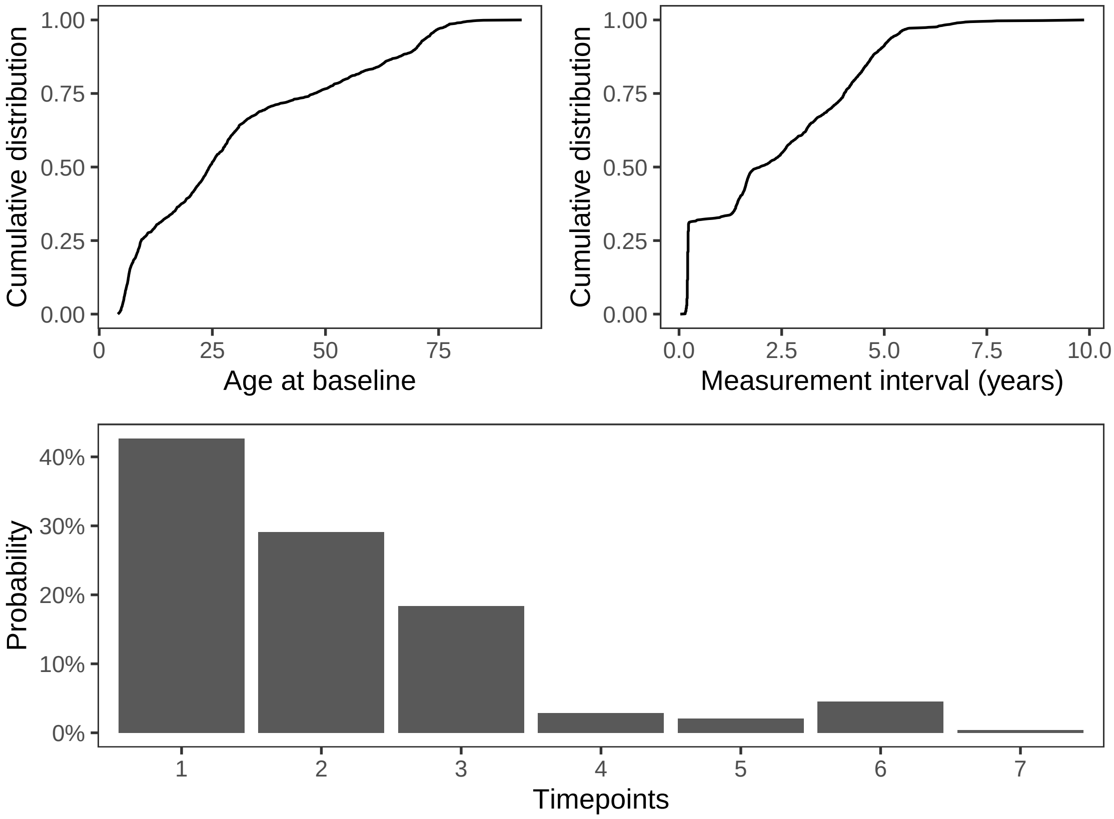

Figure S1: **Age, interval, and timepoint distributions used in simulations.** The top left plot shows the cumulative distribution of baseline age and the top right plot the cumulative distribution of times between measurements used in the simulations described in Section 6.2. The bottom plot shows the probability mass function over numbers of timepoints per participant.

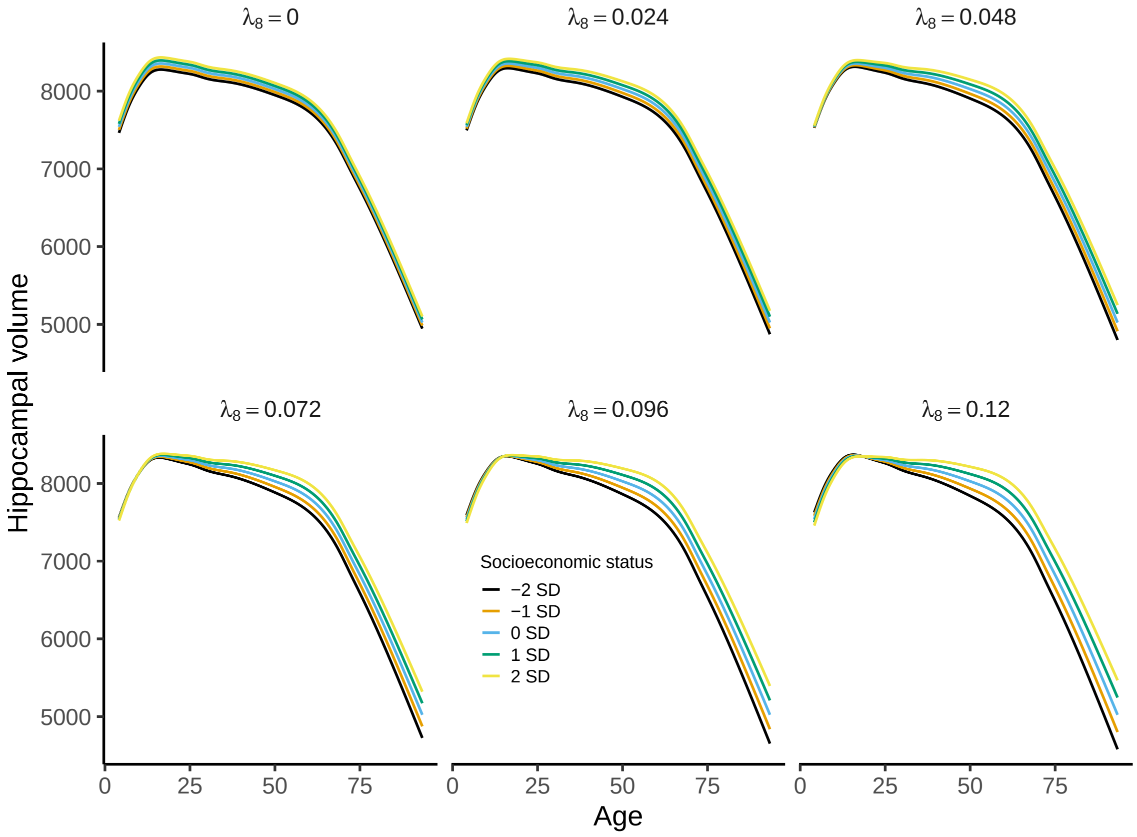

Figure S2: **Interaction values used in simulation experiments.** Visualization of the six interaction values λ_8 used in simulation experiments described in Section 6.2.

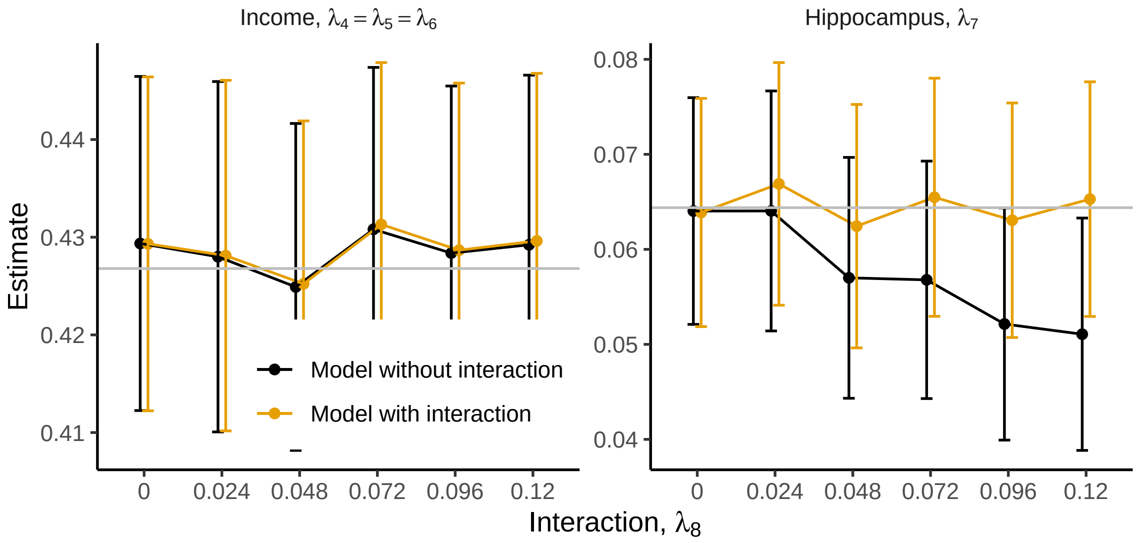

Figure S3: **Estimates of factor loadings in simulation experiments.** Estimated factor loadings in simulations with latent covariates described in Section 6.2. Horizontal gray lines show the true values of the parameter, and error bars show 95% confidence intervals due to Monte Carlo error.
